# Role of Oral Bacteria in Mediating Gemcitabine Resistance in Pancreatic Cancer

**DOI:** 10.3390/biom15071018

**Published:** 2025-07-15

**Authors:** Geng Xu, Yaling Jiang, Chen Sun, Bernd W. Brandt, Kamran Nazmi, Luca Morelli, Giulia Lencioni, Elisa Giovannetti, Dongmei Deng

**Affiliations:** 1 Department of Preventive Dentistry, Academic Center for Dentistry Amsterdam (ACTA), University of Amsterdam and VU University Amsterdam, 1081 LA Amsterdam, The Netherlands; g.xu1@amsterdamumc.nl (G.X.); y.jiang@acta.nl (Y.J.); c.sun@acta.nl (C.S.); b.brandt@acta.nl (B.W.B.); 2 Department of Medical Oncology, Cancer Center Amsterdam, Amsterdam UMC, VU University Amsterdam, 1081 HV Amsterdam, The Netherlands; e.giovannetti@amsterdamumc.nl; 3 Department of Oral Biochemistry, Academic Center for Dentistry Amsterdam (ACTA), University of Amsterdam and VU University Amsterdam, 1081 LA Amsterdam, The Netherlands; k.nazmi@acta.nl; 4 General Surgery Unit, Department of Translational Research and New Technologies in Medicine and Surgery, University of Pisa, 43, 56126 Pisa, Italy; luca.morelli@unipi.it; 5 Fondazione Pisana per la Scienza, San Giuliano Terme, 13, 56017 Pisa, Italy; g.lencioni@fpscience.it; 6 Department of Biology, University of Pisa, 43, 56126 Pisa, Italy

**Keywords:** pancreatic cancer, gemcitabine, cytidine deaminase, HPLC, drug resistance, host microbial interactions

## Abstract

Oral microbiota have been implicated in pancreatic ductal adenocarcinoma (PDAC) and may contribute to chemotherapy resistance. While previous studies attributed bacteria-induced resistance to indirect host modulation, recent findings suggest a direct mechanism. *Escherichia coli* expressing long-form cytidine deaminase (CDD_L_) can degrade gemcitabine, a chemotherapeutic agent, into a non-toxic form, leading to resistance. In contrast, bacteria carrying short form (CDD_S_) or lacking CDD did not induce resistance. This study investigates whether oral bacteria can cause gemcitabine resistance in PDAC cells through CDD-mediated degradation. Oral microbes associated with PDAC were selected based on CDD isoforms: *Aggregatibacter actinomycetemcomitans* carrying CDD_L_, *Enterococcus faecalis*, *Streptococcus mutans*, *Porphyromonas gingivalis*, all carrying CDD_S_, and *Fusobacterium nucleatum* lacking CDD. The selected microbes, along with wild-type and CDD-deficient *E. coli*, were co-incubated with gemcitabine to assess its degradation and PDAC cell proliferation. *A. actinomycetemcomitans* fully degraded gemcitabine and induced resistance. Surprisingly, CDD_S_-expressing oral bacteria partially degraded gemcitabine in a strain-dependent manner. Expressing either CDD_L_ or CDD_S_ in CDD-deficient *E. coli* resulted in equivalent gemcitabine degradation and resistance, indicating that CDD function is independent of isoform length. These findings highlight the role of oral bacteria in gemcitabine resistance and the need for strategies to mitigate microbial-driven resistance in PDAC treatment.

## 1. Introduction

Pancreatic cancer is a highly lethal malignancy, with pancreatic ductal adenocarcinoma (PDAC) accounting for approximately 90% of all cases [1]. Major risk factors include smoking, chronic pancreatitis, and a range of genetic mutations, most notably in KRAS, TP53, CDKN2A, and SMAD4 proteins, which are critical drivers of tumor initiation and progression [2]. Despite ongoing research, the global incidence of PDAC continues to rise, yet effective therapeutic options remain limited [1,2]. Currently chemotherapy is the major treatment choice, as only 10–20% of patients are eligible for surgical resection at the time of diagnosis [2,3]. Although gemcitabine/nab-paclitaxel and FOLFIRINOX regimens represent standard-of-care treatments, their clinical efficacy is often hampered by both intrinsic and acquired drug resistance [1,3].

Intrinsic mechanisms include impaired drug uptake and metabolism in pancreatic cancer cells, such as aberrations in nucleoside transporters and catabolizing enzymes [4], as well as enhanced cellular plasticity through epithelial-to-mesenchymal transition (EMT). Extrinsic influences stem largely from the tumor microenvironment (TME), which serves as both a physical barrier and an immunosuppressive niche that supports tumor survival and limits drug penetration [5]. Moreover, recent studies have identified the tumor-associated microbiome as a novel contributor to chemoresistance [6]. Ample evidence indicates that microbiota can contribute to chemoresistance, primarily through indirect mechanisms such as modulation of the host immune response and the tumor microenvironment [7]. A recent seminal study demonstrated that the microbiota can also directly influence the efficacy of chemotherapeutic agents in colorectal cancer cells (CRCs) via metabolic biotransformation. Specifically, the study revealed that bacterial species carrying the long isoform of cytidine deaminase (CDD_L_), primarily Gammaproteobacteria, deactivated gemcitabine by converting it into the inactive metabolite 2′,2′-difluorodeoxyuridine (dFdU) [8]. In contrast, bacterial species lacking CDD or carrying the short isoform of CDD (CDD_S_), except *Mycobacterium hyorhinis*, did not confer resistance to gemcitabine.

The oral microbiota is a dynamic and complex microbial community that resides in distinct sites within the oral cavity, including saliva, the tongue, oral mucosa, and tooth surfaces. While primarily localized in the oral cavity, several members of this community, such as *Porphyromonas gingivalis*, *Aggregatibacter actinomycetemcomitans*, and *Fusobacterium nucleatum*, the pathogens linked to gum diseases, have been identified in PDAC tissues, where they contributed to tumor progression [9,10,11,12]. For instance, Ilievski et al. (2020) [9] demonstrated the specific localization of *P. gingivalis* in human pancreatic α- and β-cells using 3D confocal and immunofluorescence microscopy. The prevailing view suggests oral bacteria can invade pancreatic tissues or distinct sites through hematogenous dissemination or gastrointestinal translocation [13,14,15,16,17]. In mouse models, fluorescently labeled *Enterococcus faecalis* and *Escherichia coli*, administered orally, have been tracked migrating from the oral cavity to pancreatic tissue [18]. Similarly, orally delivered *P. gingivalis* has been shown to translocate to the pancreas, where it promotes PDAC progression [19]. In addition to *P. gingivalis*, *A. actinomycetemcomitans*, *F. nucleatum*, and *Streptococci* such as *Streptococcus mutans* have also been commonly detected in the bloodstream [20,21]. These species possess virulence factors that facilitate vascular invasion and tissue colonization.

Beyond their role in carcinogenesis, oral bacteria may also influence the efficacy of chemotherapy through indirect modulation of the hosts’ immune and cellular responses [22]. For instance, *F. nucleatum* has been shown to induce resistance to 5-fluorouracil and oxaliplatin by activating the autophagy pathway and suppressing chemotherapy-induced apoptosis [23], while *P. gingivalis* mediates resistance to ceramide analog drug LCL768 in oral tumors by inhibiting ceramide-dependent mitophagy via its FimA protein [24]. However, the direct role of oral microbiota in chemoresistance, particularly in the context of PDAC, remains poorly understood. The abovementioned study of Geller et al. (2017) [8] was limited in bacterial species coverage and did not include key oral microbiota species strongly associated with PDAC. Furthermore, the relationship between these oral microbial communities and PDAC drug resistance has not been fully characterized, and the potential involvement of bacterial host regulation in CDD function remains unexplored.

Our study aims to fill these gaps by investigating the roles of specific oral microbiota in mediating gemcitabine resistance in PDAC cells through CDD-dependent degradation. Using data from the Human Oral Microbiome Database (HOMD) [25], we constructed a phylogenetic tree of CDD proteins from oral microbes. Based on their association with PDAC and the diversity of their CDD isoforms, we selected representative bacterial strains and engineered CDD-knockout and complemented *E. coli* strains. These bacterial strains were then tested for their gemcitabine-degrading activities and their influence on PDAC cell sensitivity to gemcitabine.

## 2. Materials and Methods

The in vitro study was conducted in a Biosafety Level 2 laboratory at the Academic Center for Dentistry Amsterdam. As it did not involve human participants, animals, or other regulated subjects, ethical approval was not required.

### 2.1. Cell Lines

Two PDAC cell lines, PA-TU-8988T (PATU-T, American Type Culture Collection, Manassas, VA, USA) and SUIT-2-28 (SUIT-2) [26], were routinely cultured in DMEM and RPMI-1640 media, respectively. Both media were supplemented with 10% newborn calf serum (Biowest, Nuaillé, France) and 1% penicillin-streptomycin. The cells were cultured at 37 °C in a 5% CO_2_ incubator.

### 2.2. Bacterial Strains, Plasmids, and Growth Conditions

The bacterial strains, plasmids, and primers used in this study are shown in Table 1.

*E. coli* strains were routinely cultured in Luria–Bertani broth (LB) or on solid LB media (1.5% agar) at 37 °C under aerobic conditions or at 30 °C when specified. Antibiotics were included where indicated: erythromycin (Em), 300 µg/mL, or ampicillin (Amp), 100 µg/mL, or kanamycin (Kan), 25 µg/mL.

*A. actinomycetemcomitans* Y4 was cultured in Brain Heart Infusion broth (BHI, BD Difco, Le Pont de Claix, France), supplemented with 0.02 M NaHCO_3_ and 1% glucose (BHING). *E. faecalis* V583 and *S. mutans* UA159 were cultured in BHI broth. *P. gingivalis* ATCC33277 and *F. nucleatum* ATCC10598 were cultured in BHI broth, supplemented with 5 µg/mL hemin and 1 µg/mL menadione (BHIHM). All bacterial cultures, unless otherwise specified, were grown under anaerobic conditions (80% N_2_, 10% H_2_, and 10% CO_2_) at 37 °C.

### 2.3. CDD Protein Alignment and Phylogenetic Analysis

CDD protein sequences of oral microbes were retrieved from HOMD (https://www.homd.org/ftp/genomes/PROKKA/V10.1/ accessed on 27 July 2024) [25]. After creating a non-redundant sequence set, the CDD sequences were aligned using the online Multiple Alignment using the Fast Fourier Transform (MAFFT) platform, version 7.526 [33]. Multiple alignments were performed with the FFT-NS-1 strategy (for large datasets), followed by gap reduction in MAFFT homologs using the embedded MAXalign 1.1 [34]. Realignment was carried out on the gap-free sites, after which the phylogenetic tree was constructed using the Neighbor-Joining (NJ) method [35] and visualized with ggtree (version 3.10.1, accessed on 1 July 2024) [36] in R language.

### 2.4. Construction of E. coli cdd Mutant and Complement Strains

We constructed a markerless *cdd* deletion *E. coli* strain (Eckn) to verify the function of CDD and serve as a carrier for various CDD proteins. Expression plasmids carrying the *cdd* gene from individual oral bacterial strains were transformed into Eckn, allowing for the examination of each CDD protein within this uniform microbial genetic background.

To construct the Eckn strain, we used the method described by Jensen et al. (2015) [29] and Datsenko et al. (2000) [30]. Specifically, a PCR product containing homology arms for the upstream and downstream regions of the K12 *cdd* gene, along with recombinase flippase (FLP) recognition sites (FRT) and the Kan resistance (Kan^r^) cassette from pKD4, was treated with DpnI and transformed into K12 carrying the temperature-sensitive Red helper plasmid pSIJ8. Kan^r^-positive colonies were selected and grown at 30 °C. To promote FLP-FRT recombination and remove the Kan cassette, the single Kan^r^-positive colony was grown in LB-Amp broth. The cell suspension was centrifuged, and the cell pellets were resuspended in LB-Amp broth supplemented with 0.05 M rhamnose and incubated at 30 °C for 6 h. The culture was then plated on LB-Amp plates and grown for 16 h at 30 °C. The following day, single colonies were checked by colony PCR using primers *cdd*_seq_fw/*cdd*_seq_rv (Table 1) to verify the removal of Kan cassette. Plasmid pSIJ8 was cured by growing the cells without antibiotics at 37 °C. The *cdd* deletion in K12 was confirmed by Sanger sequencing.

To construct various Eckn strains carrying the *cdd* expression vector, the low-copy number shuttle plasmid pVA838 was used as a backbone. The constitutive P32 promoter was restricted from pMG36e [32], and the *cdd* genes from various bacterial strains were amplified by PCR using the primer sets listed in Table 1. The P32 promoter and the individual *cdd* genes were inserted in pVA838, resulting in a set of new plasmids, listed in Table 1. Each plasmid was transformed into the Eckn strain, resulting in four different strains. The sequences of the *cdd* genes were verified by Sanger sequencing.

### 2.5. Bacteria and Gemcitabine Co-Incubation

Bacteria-mediated gemcitabine resistance was examined following the procedure outlined by Geller et al. (2017) [8], with modifications. Various bacterial strains, including wild-type strains, such as *E. coli* and *A. actinomycetemcomitans*, Eckn, and complement strains including Eckn carrying various *cdd* genes, were cultured for 16–24 h. The full-grown culture was centrifuged (16,800 rpm, 2 min), and the bacterial cell pellets were resuspended in Phosphate Buffered Saline (PBS), without antibiotics, to achieve an optical density (OD600) of 1, equivalent to approximately 10^8^ CFU/mL bacterial cells. Gemcitabine was then added to the bacterial suspension at a final concentration of 10 µM. Milli-Q was included as the negative control. The bacteria–gemcitabine mixtures were incubated at 37 °C under aerobic conditions or anaerobically (for *P. gingivalis*) for 1 or 4 h, then filtered through 0.2 µm sterile membrane filters (Sarstedt, Nümbrecht, Germany). The filtrates were aliquoted and stored at −20 °C for further analysis, including PDAC cell drug inhibition assay and quantifications of dFdC and dFdU.

### 2.6. PDAC Cell Drug Inhibition Assay

The effect of bacteria–gemcitabine filtrates on PDAC cell growth was assessed using PATU-T and SUIT-2 cell lines. Both cell lines were seeded in triplicate at a density of 3000 cells per well in 96-well plates and allowed to attach for 24 h. Serial dilutions of the bacteria–gemcitabine filtrates were added to the wells, with a 10 µM gemcitabine solution prepared and diluted in the same manner as the positive control. After 72 h of growth, the cell proteins were precipitated and stained with sulforhodamine B (SRB). The stained precipitations were resuspended in 10 mM Tris buffer, and the absorbance was measured at 492 nm using the SpectraMax^®^ i3x Multi-Mode Microplate Reader (Molecular Devices, Silicon Valley, CA, USA) [37]. The inhibitory concentration required to reduce cell quantity by 50% (IC50) was determined by nonlinear least-squares curve fitting, using Graphpad Prism (version 8.0, Intuitive Software for Science, San Diego, CA, USA).

### 2.7. Quantifications of dFdC/dFdU in Bacteria–Gemcitabine Filtrates

The concentrations of dFdC and dFdU in the bacteria–gemcitabine filtrates were quantified using an in-house HPLC protocol. Briefly, the filtrates were analyzed in an ion-pair reversed-phase Ultimate 3000 HPLC system (Dionex, Sunnyvale, CA, USA) equipped with a Symmetry C18 column (3.9 mm × 150 mm, Waters, Milford, CT, USA). The running buffer consisted of 1% PIC-B7 (heptane sulfonic acid solution) and 3% acetonitrile, adjusted to pH 2.8 with hydrogen chloride. The flow rate was set at 1.0 mL/min, with UV detection at 254 and 280 nm. The injection volume was 70 µL, and each sample was analyzed for 15 min. HPLC data were recorded and processed using Chromeleon software (Version 7.3, ThermoFisher, Waltham, MA, USA). A calibration curve was constructed from standard samples containing 0.5 µM to 150 µM dFdC and 0.5 µM to 200 µM dFdU. The concentrations of dFdC and dFdU in each sample were determined using Pearson linear regression based on the peak areas under the curves [38]. The proportion of dFdC and dFdU in each sample was calculated as the ratio of the concentration of dFdC or dFdU to the total concentration of both.

### 2.8. Statistical Analysis

Data analysis was conducted using GraphPad Prism (version 8.0, Intuitive Software for Science, San Diego, CA, USA), and graphs were generated using GraphPad Prism. The normality of data distribution was confirmed using the Shapiro–Wilk test. Results are reported as mean ± standard deviation (SD). For statistical comparisons, data from the 1 h (IC50 values and proportions of dFdC/dFdU) and 4 h (IC50 values) bacteria–gemcitabine co-incubation were analyzed separately. One-way ANOVA was applied to each dataset to assess difference between bacterial strains, followed by Dunnett’s post hoc test. A *p*-value of <0.05 was considered statistically significant. All experiments were conducted in triplicate, with 2–3 technical replicates per experiment.

## 3. Results

### 3.1. Alignment of CDD Protein Sequences

First, we investigated whether bacteria were present in pancreatic cancer tissues. Figure S1a shows histological sections of pancreatic cancer from patients. The fluorescent image in Figure S1b demonstrates our detection of bacterial presence in pancreatic cancer using specific probes, with red labeling indicating bacteria.

**Figure 1:**
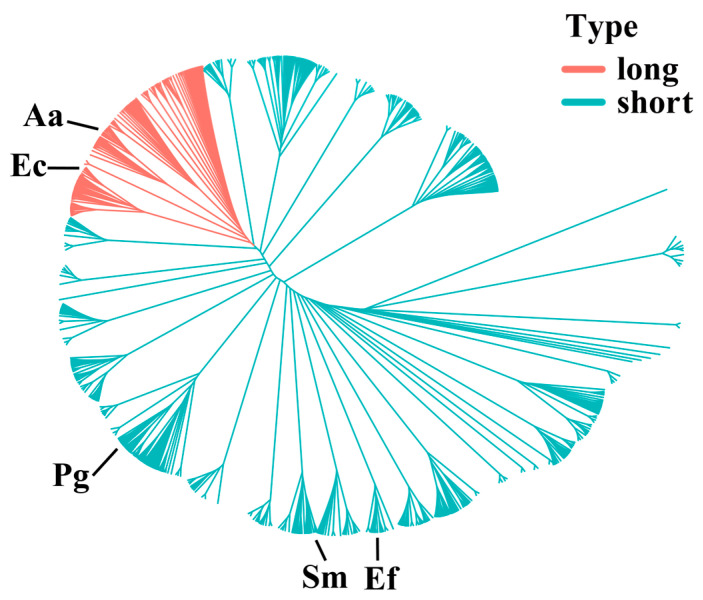
The phylogenetic tree of CDD proteins was constructed utilizing 86 gap-free sites. The red lines indicate the cluster of long isoform CDD proteins, which span from 250 to 360 amino acids in length. The green lines indicate the cluster of short isoform CDD proteins. Ec refers to *E. coli* K12 (294 AA), Aa to *A. actinomycetemcomitans* Y4 (298 AA), Pg to *P. gingivalis* ATCC33277 (158 AA), Ef to *E. faecalis* V538 (131 AA), and Sm to *S. mutans* UA159 (144 AA).

A total of 1366 CDD protein sequences remained after retrieving all known CDD sequences from the HOMD database and removing duplicates. The lengths of these CDD protein sequences varied considerably, ranging from 61 to 330 amino acids (AA). The initial sequence alignment did not yield any gap-free sites. To address this, MAXalign was used to remove badly aligned or truncated sequences, after which 1204 sequences remained. The alignment of these sequences identified 86 gap-free aligned sites, which were used to construct a phylogenetic tree (Figure 1). This tree provides an overview of the evolutionary relationships among CDD proteins across oral bacteria. The red region highlights a cluster of CDD_L_ proteins, ranging from 250 to 360 AA in length, which form a distinct group separate from the CDD_S_ proteins. The CDD_S_ exhibits a large number of branches, indicating a high degree of sequence divergence. Given this divergence, we speculate that the CDD_S_ may possess some cytidine-deaminase activity.

Based on the reported association with PDAC [39], two bacterial strains containing CDD_L_—*E. coli* K12 (294 AA) and *A. actinomycetemcomitans* Y4 (298 AA); three strains containing CDD_S_—*Streptococcus mutans* UA159 (144 AA), *Enterococcus faecalis* V583 (131 AA), and *P. gingivalis* ATCC33277 (158 AA); and one strain containing no CDD—*F. nucleatum*—were selected for further analysis. The protein lengths (in AA) are indicated in parentheses.

### 3.2. Growth Inhibition of PDAC Cells by Bacteria–Gemcitabine Filtrates

**Figure 2:**
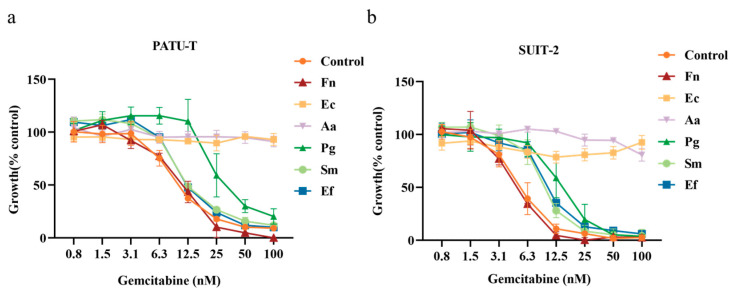
Inhibition of cell proliferation in PATU-T (**a**) and SUIT-2 (**b**) cells by bacteria–gemcitabine filtrates. Bacterial cultures were incubated with 10 µM gemcitabine for 1 h, and, for *F. nucleatum*, *S. mutants*, and *E. faecalis*, the co-incubation duration was extended to 4 h. The resulting bacteria–gemcitabine filtrates were examined for their growth inhibitory effects on PDAC cells by SRB assay. Fn: *F. nucleatum*; Ec: *E.coli*; Aa: *A. actinomycetemcomitans*; Pg: *P. gingivalis*; Sm: *S. mutans*; Ef: *E. faecalis*.

Bacterial cultures were incubated with 10 µM gemcitabine for 1 h, and, for *F. nucleatum*, *S. mutants,* and *E. faecalis*, the co-incubation duration was extended to 4 h. The resulting bacteria–gemcitabine filtrates were examined for their growth inhibitory effects on PDAC cells. Figure 2a,b shows that the positive control group, serial dilutions of gemcitabine alone, exhibited a dose-dependent growth inhibition. In contrast, bacteria–gemcitabine filtrates displayed strain-dependent inhibition patterns. IC50 values calculated from the growth inhibition curves are presented in Table 2.

For *F. nucleatum*, extending the co-incubation time to 4 h did not affect the gemcitabine inhibition pattern. However, co-incubation with *E. coli* and *A. actinomycetemcomitans*, carrying long-form CDD for 1 h completely abolished gemcitabine’s inhibitory effects across both tested cell lines. Surprisingly, co-incubation with strains carrying short-form CDD significantly reduced gemcitabine efficacy, although the extent varied by strain. For *P. gingivalis*, a 1 h co-incubation markedly reduced growth inhibition, with a stronger effect on PATU-T cells compared to SUIT-2 cells. In the case of *E. faecalis*, a 1 h co-incubation significantly reduced gemcitabine’s efficacy in SUIT-2 cells. For *S. mutans*, this reduction was significant only after 4 h of co-incubation.

### 3.3. Quantification of Gemcitabine Degradation in Filtrates

**Figure 3:**
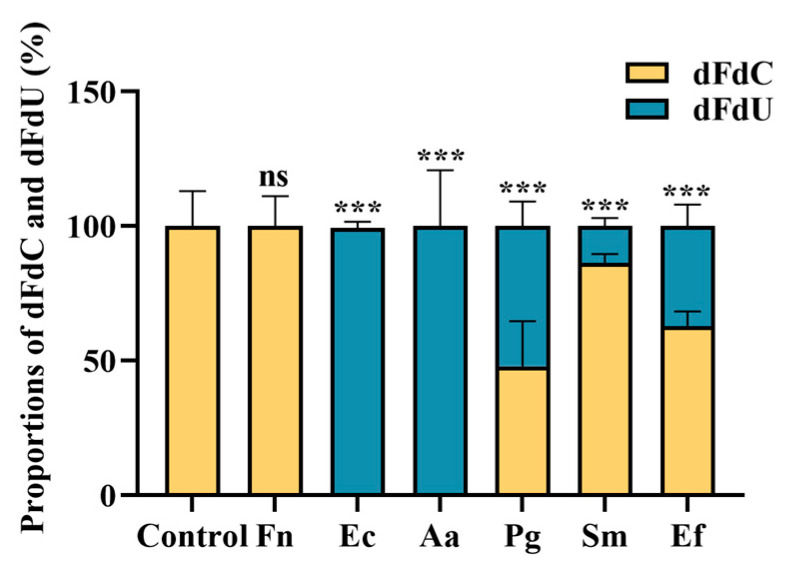
Proportions of gemcitabine (dFdC) and dFdU in bacteria–gemcitabine filtrates. HPLC quantified the concentrations of dFdC and dFdU in bacteria–gemcitabine filtrates after the bacteria mixed with 10 µM gemcitabine for 1 h. Fn: *F. nucleatum*; Ec: *E. coli*; Aa: *A. actinomycetemcomitans*; Pg: *P. gingivalis*; Sm: *S. mutans*; Ef: *E. faecalis*. The statistical significance of differences in dFdC between the control group and other group (s) was defined as follows: ns (not significant) for *p* > 0.05; *** for *p* < 0.001.

To explain the reduction in gemcitabine efficacy observed with different bacterial strains, we quantified the concentrations of gemcitabine (dFdC) and its CDD-metabolized product (dFdU) in the filtrates using HPLC. Figure 3 shows that filtrates from gemcitabine alone and gemcitabine–*F. nucleatum* contained only dFdC, with no detectable dFdU. In contrast, filtrates from *E. coli* and *A. actinomycetemcomitans* contained only dFdU. Other bacterial filtrates contained varying proportions of dFdC and dFdU, with the highest proportion of dFdU relative to dFdC detected in *P. gingivalis* filtrates and the lowest in *S. mutans*. These data suggest that the reduction in gemcitabine efficacy is due to the degradation of dFdC to dFdU, likely mediated by bacterial CDD. Bacteria containing CDD_S_ are capable of breaking down gemcitabine but are less potent than those carrying CDD_L_.

### 3.4. CDD Function Is Independent of CDD Length

**Figure 4:**
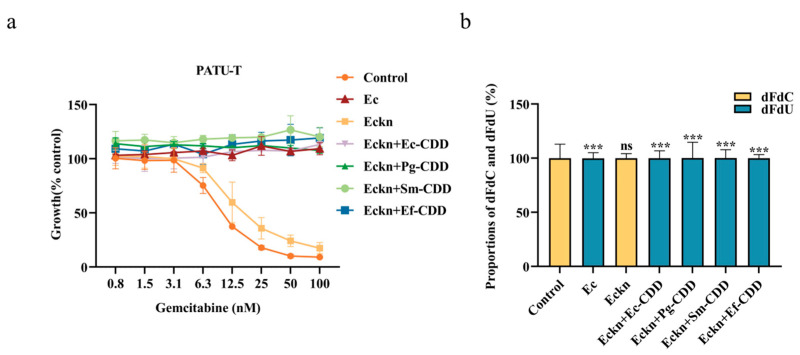
Assessment of CDD protein activity in bacteria–gemcitabine filtrates based on PATU-T cell growth inhibition and gemcitabine degradation. (**a**) The SRB assay demonstrated the inhibitory effect of bacterial filtrates of gemcitabine and different bacteria on PATU-T growth. (**b**) HPLC quantified the concentrations of dFdC and dFdU in bacteria–gemcitabine filtrates after the bacteria mixed with 10 µM gemcitabine for 1 h. Ec: *E. coli*; Eckn: *cdd* deletion *E. coli* strain; Eckn + Ec-CDD: complementing Eckn with CDD originating from *E. coli*; Eckn + Pg-CDD, Eckn + Sm-CDD and Eckn + Ef-CDD: complementing Eckn with CDD originating from *P. gingivalis*, *S. mutans*, and *E. faecalis*. The statistical significance of differences in dFdC between the control group and other group (s) was defined as follows: ns (not significant) for *p* > 0.05; *** for *p* < 0.001.

To confirm the role of bacterial CDD in dFdC degradation and clarify functional differences among different CDDs, we constructed an *E. coli* strain lacking the *cdd* gene (Eckn) and complemented it with plasmids encoding CDD_S_ from *P. gingivalis*, *S. mutans*, and *E. faecalis*. As shown in Figure 4a, filtrates from wild-type *E. coli* completely abolished gemcitabine’s inhibitory effects on PATU-T cells, while filtrates from the Eckn strain did not, behaving similarly to gemcitabine alone. Introduction of the plasmid encoding Ec-CDD_L_ into Eckn fully restored gemcitabine resistance. Surprisingly, plasmids encoding CDD_S_ from other bacterial strains also restored 100% gemcitabine resistance.

HPLC analysis confirmed that dFdC was entirely converted to non-cytotoxic dFdU in filtrates from both wild-type *E. coli* and Eckn strains carrying CDD plas mids (Figure 4b). These findings demonstrate that the functionality of CDD in gemcitabine degradation is independent of protein length, with both long-form and short-form CDD being equally effective in dFdC breakdown.

## 4. Discussion

It has been shown that *E. coli* can confer gemcitabine resistance in CRC cells by converting gemcitabine into the non-cytotoxic dFdU via the long isoform of CDD [8]. The study concluded that “Bacteria that either lack CDD or express the short isoform of the enzyme (CDD_S_) were unable to mediate resistance to gemcitabine” [8,40]. The current study confirmed some of these previous findings, demonstrating that oral bacteria expressing CDD_L_ can induce gemcitabine resistance in PDAC cells, while those lacking CDD cannot. However, in contrast to the previous claim, our data reveal that bacteria carrying CDD_S_ can partially confer gemcitabine resistance in a species-dependent manner. This functional variation cannot be explained by the length or origin of the short-form CDD proteins.

It was surprising that the bacteria expressing CDD_S_ were able to partially confer gemcitabine resistance in PDAC cells in our experimental setting, which contradicts previous conclusions regarding CDD_S_. Among the CDD_S_-expressing bacterial strains examined, *E. faecalis* V583 was also tested in Geller et al. (2017) [8], where no breakdown of dFdC was observed. This discrepancy may be explained by differences in the methods used for dFdC quantification. Geller’s study applied 4 µM dFdC to the bacterial culture and quantified the remaining dFdC in the filtrates, whereas our study used 10 µM dFdC and measured both the remaining dFdC and the metabolite dFdU. It has been reported that the sensitivity range of the HPLC method for gemcitabine quantification is between 1 µM and 200 µM [41], which may have limited the detection of partial dFdC reduction in Geller’s study. In contrast, the higher initial dFdC concentration in our study, along with the detection of both dFdC and dFdU, enabled us to detect the function of CDD_S_ more effectively.

Interestingly, our data show that all *cdd* homologs were able to restore CDD function in the *E. coli cdd* null mutant, irrespective of CDD protein length. The expressed CDD_S_ protein demonstrated the same function as the CDD_L_ protein in gemcitabine degradation and the restoration of gemcitabine resistance in PDAC cells, despite a significant sequence divergence among CDD_S_ proteins.

Adding to the complexity, despite the expressed CDD_S_ showing the same function as CDD_L_ proteins, the CDD_S_ variants exhibited varying dFdC breakdown activities in their original hosts. This observation suggests that CDD functionality is influenced not only by its isoform length but also by host-specific regulatory mechanisms. In bacterial systems, gene expression can be modulated by intrinsic factors such as transcriptional regulators, sigma factors, and promoter accessibility, all of which respond to environmental cues and cellular conditions [42,43,44]. Additionally, metabolic byproducts may exert feedback regulation on gene expression, with accumulated intermediates acting as signaling molecules that influence transcription factor activity [44,45,46]. Comparable phenomena are observed in other bacterial proteins, such as Hfq, an RNA-binding protein that facilitates interaction between small RNAs (sRNAs) and their trans-encoded mRNAs targets [47]. Notably, Hfq binds sRNAs tightly in Gram-negative bacteria like *E. coli* [48] but exhibits reduced functionality in Gram-positive species such as *Staphylococcus aureus*. These functional differences have been attributed to both structural features and divergent regulatory system differences [49]. Similarly, the weaker gemcitabine degradation activity observed in Gram-positive *S. mutans* and *E. faecalis*, compared to the more efficient activity in Gram-negative *P. gingivalis*, is likely multifactorial. Structural disparities in the CDD_S_ proteins, along with differences in host regulatory networks, may collectively contribute to the observed functional variability. Further investigation into these structural and regulatory elements will be essential for understanding the mechanistic underpinnings of host-specific CDD_S_ activity and could ultimately inform microbiota-targeted strategies to improve gemcitabine efficacy.

The evidence presented in this study strongly suggests that a broader range of bacterial strains than previously recognized may contribute to gemcitabine resistance in PDAC. However, a critical question remains: are these bacteria present along the gemcitabine delivery route in vivo? In addition to previously reported evidence of oral bacteria in the blood stream and within PDAC tissues [9,10,11,12,49]. We demonstrated bacterial presence in PDAC tumor tissues using a Cy5-labelled universal fluorescence in situ hybridization probe (see Supplementary Materials). Collectively, these findings underscore the potential for microbiota in directly compromising the efficacy of chemotherapeutic agents. Further research is warranted to elucidate this mechanism and to develop therapeutic strategies that target microbiota-mediated chemoresistance.

## 5. Conclusions

In contrast to previous studies which suggested that only CDD_L_ could degrade gemcitabine, our data demonstrated that bacterial CDD proteins—regardless of isoform length—are capable of conferring chemoresistance by converting gemcitabine into its non-toxic metabolite dFdU. Furthermore, we discovered that the bacterial strain in question can influence and regulate CDD’s degradation capacity. These findings offer novel perspectives on the mechanistic role of bacterial CDD in mediating gemcitabine resistance in PDAC, and they raise important new questions about how bacterial host factors may modulate CDD activity.

## Figures and Tables

**Table 1:** Bacterial strains, plasmids, and primers used in this study.

Strains	Relevant Characteristic(s)	Source of Reference
*E. coli* K12	Wild type	DSMZ
*E. coli* DH5α	F-, endA1deoR, recA1, hsdR17 (rk-,mk+), supE44, thi-1, gyrA96, relA1	Invitrogen
*A. actinomycetem-comitans* Y4	Wild type	Our own strain collection
*S. mutans* UA159	Wild type	(Ajdić et al., 2002) [27]
*E. faecalis* V583	Wild type	(Duggan and Sedgley, 2007) [28]
*P. gingivalis* ATCC33277	Wild type	ATCC
*F. nucleatum* ATCC10953	Wild type	ATCC
*E. coli* Δ*cdd* (Eckn)	*E. coli* K12 with deletion of *cdd*	This study
**Plasmids**		
pSIJ8	Temperature-sensitive plasmid containing the lambda Red recombineering genes and a flippase recombinase, Amp^r^	(Jensen et al., 2015) [29]
pKD4	*E. coli* knockout vector, Amp^r^, Kan^r^	(Datsenko and Wanner, 2000) [30]
pVA838	*E. coli*-streptococcal shuttle vector; E^r^	(Macrina et al., 1982) [31]
pMG36e	*E. coli*-lactococcal expression vector, containing constitutive lactococcal P32 promoter; Em^r^	(van de Guchte et al., 1989) [32]
pJV1	pVA838 containing P32 and *cdd* gene from *E. coli* K12	This study
pJV2	pVA838 containing P32 and *cdd* gene from *S. mutans* UA159	This study
pJV9	pVA838 containing P32 and *cdd* gene from *E. faecalis* V583	This study
pJV12	pVA838 containing P32 and *cdd* gene from *P. gingivalis* ATCC33277	This study
**Primers**	Oligonucleotide and sequence (5′ to 3′)	
*cdd*_fw	ACGGGTTCGTAAACTGTTATCCCATTACATGATTATGAGGCAACGCCATGTGTAGGCTGGAGCTGCTTC	
*cdd*_rv	AAGGCGTTCACGCCGCATCCGGCACCAGGCTTAAGCGAGAAGCACTCGGTGCTTGCATATGAATATCCTCCTTAG	
*cdd*_seq_fw	CCGAGCTGGATTATCAGGAAGG	
*cdd*_seq_rv	GGACTAACAGGCTGAGGAACAC	
*cdd*_Ec_fw	ATGCATCCACGTTTTCAAACC	
*cdd*_Ec_rv	TTAAGCGAGAAGCACTCGGTCGAT	
*cdd*_Sm_fw	ATGGTGGTTATTGATTTAATCAG	
*cdd*_Sm_rv	TTACTTTAACTCTCTAAAGGAATAAG	
*cdd*_Ef_fw	ATGACAGTAAAACAAGAATGGCTTGAT	
*cdd*_Ef_rv	TTAAAAATCTTTATCTGTAAATG	
*cdd*_Pg_fw	ATGCTCCGTAAAACTCTTTC	
*cdd*_Pg_rv	CTATCGTTCCAAGTCGCTACCG	

pSIJ8 was purchased from Addgene (plasmid # 68122); pKD4 was purchased from Addgene (plasmid # 45605). The primers contain homology arms of up- or downstream sequences of *E. coli* K12 *cdd* and 20-nt priming sequences for pKD4 (underlined). Ec: *E. coli*; Sm: *S. mutans*; Ef: *E. faecalis*; Pg: *P. gingivalis.*

**Table 2:** IC50 values (nM) of gemcitabine and gemcitabine–bacteria filtrates on PATU-T and SUIT-2 cell lines.

Tested Samples	PATU-T		SUIT-2	
	1 h	4 h	1 h	4 h
Gem	11.6 ± 1.6		5.1 ± 2.1	
Gem-Fn	11.9 ± 0.6	11.1 ± 1.1	4.9 ± 1.3	5.1 ± 0.8
Gem-Ec	NI	–	NI	–
Gem-Aa	NI	–	NI	–
Gem-Pg	51.1 ± 18.4 ***	–	15.5 ± 4.3 ***	–
Gem-Ef	17.3 ± 2.1	17.1 ± 1.1 **	10.1 ± 3.6 *	11.4 ± 1.7 ***
Gem-Sm	15.2 ± 0.2	18.1 ± 1.7 **	8.6 ± 3.1	10.8 ± 1.6 ***

IC50: the inhibitory concentration required to reduce cell quantity by 50%. Gem: gemcitabine; Fn: *F. nucleatum*; Ec: wild-type *E. coli*; Aa: *A. actinomycetemcomitans*; Pg: *P. gingivalis*; Sm: *S. mutans*; Ef: *E. faecalis*; “–”: not measured; NI: no growth inhibition. The statistical significance of differences between the Gem group and other group (s) was defined as follows: * for *p* < 0.05; ** for *p* < 0.01; *** for *p* < 0.001.

## Data Availability

The datasets used and/or analyzed during the current study are available from the corresponding author on reasonable request.

## Supplementary Materials

The following supporting information can be downloaded at: https://www.mdpi.com/article/10.3390/biom15071018/s1, Figure S1. Detection of Bacterial Presence in PDAC Tissues [50,51].

## Abbreviations

PDAC	pancreatic ductal adenocarcinoma
CDD	cytidine deaminase
CDD_L_	long isoform of CDD
CDD_S_	short isoform of CDD
PATU-T	PA-TU-8988T
SUIT-2	SUIT-2-28
SRB	sulforhodamine B
Eckn	*cdd* deletion *E. coli* strain
