# Supplementary material for: Role of Oral Bacteria in Mediating Gemcitabine Resistance in Pancreatic Cancer
Source: Biomolecules. 2025 Jul 15;15(7):1018. doi: 10.3390/biom15071018 (PMC12293802; doi:10.3390/biom15071018)
Supplement: Supplementary file 1 [file biomolecules-15-01018-s001.zip › biomolecules-3680640-supplementary.pdf]

Supplementary materials

1. Clinical sample

PDAC tissue samples were obtained from patients enrolled at Pisa University Hospital, following approval by the Ethics Committee of Area Vasta Nord Ovest (CEAVNO, protocol #724). PDAC cases were validated through comprehensive clinical-pathological evaluation in accordance with the revised international cancer staging system [50].

2. Hematoxylin and Eosin staining (H&E)

Hematoxylin and Eosin staining was conducted on resected PDAC samples. We used the procedure described by a previous study [51]. The images were acquired using the Euromex CMEX 5.0 camera software on the Olympus BX50 microscope (Tokyo, Japan).

3. Fluorescence in situ Hybridization (FISH) for detecting tissue-associated bacteria

To detect bacterial presence in tumor tissue, a Cy5-labeled universal bacterial probe, the Ribo Technologies FISH Kit (BioVisible, Groningen, the Netherlands), was used.

Formalin-fixed, paraffin-embedded tissue sections were first deparaffinized in xylene, then rehydrated through a graded ethanol series to 100% ethanol. Hybridization was performed using the pre-warmed (50 °C) probe in FISH hybridization buffer. Coverslips were applied, and the sections were incubated overnight at 50 °C in a dark and humidified chamber. Following hybridization, the slides were washed in a pre-heated diluted washing buffer at 50 °C for 30 minutes, then rinsed in deionized water for another 30 minutes. The slides were dried in the dark and mounted using the antifade mounting medium provided in the kit. The coverslips were sealed with nail polish to preserve fluorescence.

Images were acquired using a confocal microscope (Axio Vert 200, Carl Zeiss Microscopy, Jena, Germany) equipped with dedicated imaging software. Fluorescence was detected using an excitation wavelength of 642 nm and an emission wavelength of 670 nm under 100× magnification.

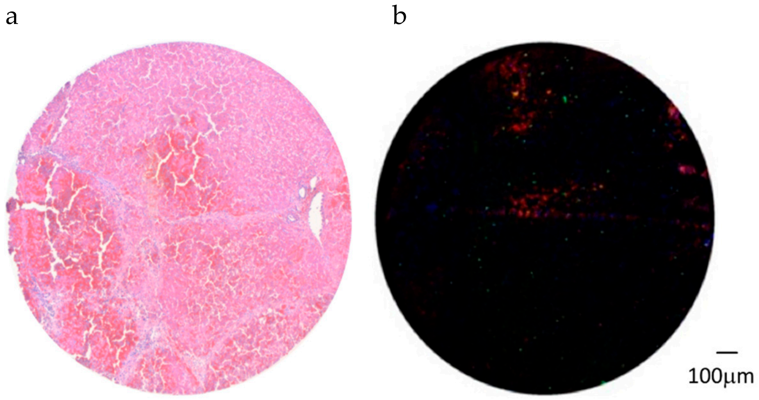

Figure S1. Detection of bacterial presence in PDAC tissues. (a) Representative H&E-stained image of a PDAC tumor section. (b) Fluorescence in situ hybridization using a Cy5-labeled universal bacterial probe to detect bacterial presence in PDAC tissue. Red color: Cy5; other color: background colors caused by the autofluorescence of the tissue.
